# Spatiotemporal Patterns of Indoor Air Pollution and Its Association with Depressive Symptoms Among Schoolchildren in China

**DOI:** 10.3390/toxics13070563

**Published:** 2025-07-01

**Authors:** Yaqi Wang, Di Shi, Xinyao Ye, Jiajia Dang, Jianhui Guo, Xinyao Lian, Shaoguan Wang, Jieyun Song, Yanhui Dong, Jing Li, Yi Song

**Affiliations:** 1 Institute of Child and Adolescent Health, School of Public Health, Peking University, Beijing 100191, China; 2211110227@bjmu.edu.cn (Y.W.); 2111210123@bjmu.edu.cn (D.S.); dangjj@bjmu.edu.cn (J.D.); gjh201001@163.com (J.G.); a987268196@163.com (X.L.); 15878641416@163.com (S.W.); songjieyun1983@126.com (J.S.); dongyanhui@bjmu.edu.cn (Y.D.); songyi@bjmu.edu.cn (Y.S.); 2 National Health Commission Key Laboratory of Reproductive Health, Peking University, Beijing 100191, China; 3 Department of Epidemiology and Biostatistics, Public Health School, Peking University, Beijing 100191, China; 2010306208@stu.pku.edu.cn

**Keywords:** indoor air pollution, particulate matter, carbon dioxide, formaldehyde, total volatile organic compounds, depressive symptoms

## Abstract

Despite spending a substantial proportion of their time indoors, the mental health effects of indoor air pollution on children and adolescents remain inadequately explored. This study aimed to elucidate the spatiotemporal variations and sociodemographic inequalities in exposure to multiple indoor pollutants and to assess their potential associations with depressive symptoms among school-aged children in Beijing. Using real-time portable monitors, concentrations of fine particulate matter (PM_2.5_), coarse particulate matter (PM_10_), carbon dioxide (CO_2_), formaldehyde (HCHO), total volatile organic compounds (TVOC), temperature, and humidity in classrooms and bedrooms were measured during both weekdays and weekends. Moreover, substantial spatiotemporal heterogeneity was observed. It was found that concentrations of PM_2.5_, PM_10_, and TVOC peaked in classrooms during weekday daytime, while CO_2_ levels were highest in bedrooms on weekend nights. Exposure levels were notably higher among children whose mothers had lower educational attainment and those living in recently renovated homes, indicating marked socio-demographic disparities. In multivariable logistic regression models, indoor exposure to CO_2_ and TVOC was significantly associated with increased odds of depressive symptoms. These findings highlight the critical need to improve indoor air quality through enhanced ventilation and the mitigation of emissions from indoor sources, particularly within school and residential settings. The results offer valuable empirical evidence to guide the development of targeted environmental interventions and public health policies designed to support and enhance the psychological well-being of children.

## 1. Introduction

Children and adolescents spend a substantial proportion of their time indoors, particularly in urban settings where indoor environments account for more than 80% of daily activities [1,2]. Accordingly, indoor air quality (IAQ) constitutes a critical determinant of health for this population. Compared to adults, children and adolescents are more vulnerable to indoor air pollution due to their higher respiratory rates, developing immune systems, and greater exposure relative to body weight [3]. Moreover, the prolonged time they spend in classrooms and bedrooms further elevates both the duration and intensity of exposure to indoor pollutants. Indoor environments contain a complex mixture of pollutants, including particulate matter, carbon dioxide (CO_2_), total volatile organic compounds (TVOC), and formaldehyde (HCHO). All of these exposures have been implicated in a range of adverse health outcomes, most notably respiratory diseases [4] and cardiovascular effects [5]. Furthermore, exposure to specific indoor pollutants, including elevated CO_2_, which reflects inadequate ventilation, and TVOCs, which have potential neurotoxic and endocrine-disrupting properties, has been associated with adverse cognitive outcomes such as reduced attention and impaired learning, as well as behavioral problems including hyperactivity and conduct issues in children [6]. These effects may co-occur with, or contribute to, broader mental health concerns. Accurately assessing pollutant exposure in the daily microenvironments of children and adolescents is therefore essential for identifying health risks and informing targeted intervention strategies.

While the adverse effects of indoor air pollution on physical, cognitive, and behavioral health are increasingly recognized, its associations with psychological well-being, especially the development of depressive symptoms in children and adolescents, remain insufficiently understood. In contrast, epidemiological studies have linked outdoor air pollution, especially fine particulate matter, to elevated risks of depression and anxiety in young populations [7,8], highlighting the need to explore whether similar associations exist for indoor exposures. Many previous studies have relied on short-term measurements taken from a single indoor environment [9,10,11], most commonly the home, and have not adequately captured the temporal and spatial variability of exposure across key settings such as classrooms and bedrooms. Furthermore, existing research has often focused on a narrow set of pollutants, with limited consideration of the combined effects of multiple exposures. Important indicators such as CO_2_, which reflects ventilation adequacy [12], and TVOCs, which may exert neurotoxic or endocrine-disrupting effects [13,14], have received insufficient attention.

To address these gaps, the present study employed real-time portable monitors to measure seven key indoor environmental parameters in both school and home settings during weekdays and weekends. These parameters included particulate matter with an aerodynamic diameter of ≤2.5 μm (PM_2.5_), particulate matter with an aerodynamic diameter of ≤10 μm (PM_10_), CO_2_, HCHO, TVOC, indoor temperature, and relative humidity. Exceedance rates for these pollutants were evaluated based on the thresholds set by Indoor Air Quality Standard of China (GB/T 18883–2022) [15], which provides health-based limits widely used for IAQ assessment in Chinese populations. The study aimed to characterize the spatiotemporal distribution and exceedance rates of these pollutants, investigate sociodemographic disparities in exposure levels, and evaluate the associations between indoor pollutant concentrations and depressive symptoms among children and adolescents. By adopting a comprehensive framework that simultaneously considered multiple pollutants across varied indoor environments, this research provides novel insights into real-world exposure profiles and identifies critical environmental determinants potentially influencing pediatric mental health. These findings contribute a valuable empirical foundation for the development of targeted public health interventions aimed at enhancing IAQ and promoting the psychological well-being of school-aged populations.

## 2. Materials and Methods

### 2.1. Participants

Participants were recruited through convenience sampling from grades 1–4 and grade 7 within the natural control group of a school-based intervention survey. Students in grades 5–6 and 8–9 were excluded due to anticipated attrition and increased academic demands during the follow-up period, as outlined in the published study protocol [16]. The survey was conducted across three districts in Beijing, China, including Dongcheng, Tongzhou, and Changping. A total of 235 children and adolescents, aged 6 to 14 years, from 18 schools were enrolled to assess their exposure to indoor air pollutants between March and December 2023. Informed consent was obtained from the parents or legal guardians of all participants. The study protocol was approved by the Ethics Review Committee of Peking University Health Science Center (Approval No. 00001052-22018).

### 2.2. Measurements of Indoor Exposure to Air Pollutants

Yunhe QD-G3 portable monitors (Beijing Yunhe Healthy Habitat Technology Co., Ltd., Beijing, China) were employed to continuously measure indoor concentrations of PM_2.5_, PM_10_, CO_2_, HCHO, TVOC, temperature, and humidity over a seven-day monitoring period. Detailed technical specifications of the monitoring devices are provided in Table S1. Measurements were conducted continuously for 24 h per day over five weekdays (Monday to Friday) and two weekend days (Saturday and Sunday). Indoor data were collected from two distinct locations, including participants’ bedrooms and classrooms. Under parental supervision, the portable monitors were placed on bedroom tables at approximately breathing zone height to ensure unobstructed sensor function and to reflect typical indoor exposure. Similarly, in classrooms, the devices were positioned on desks at the students’ breathing zone height to accurately capture daytime exposure levels. The monitors recorded pollutant concentrations at five-minute intervals from 8:00 a.m. to 8:00 a.m. the following day.

To ensure measurement accuracy, all Yunhe QD-G3 devices were calibrated prior to field deployment. PM_2.5_ sensors were calibrated against a TSI DustTrak DRX 8530 aerosol monitor, and CO_2_ sensors were validated using a TSI IAQ-Calc 7515 instrument, both following the T/CECS 698-2020 standard [17]. For HCHO, TVOC, temperature, and humidity, device-specific correction equations were derived from multi-point comparisons in a controlled environmental chamber with reference concentrations. In addition, monthly zero-calibration using HEPA filters (for PM) and clean air (for gases) was conducted throughout the study period. Duplicate deployments and daily plausibility checks were also performed to ensure ongoing data quality.

Data were considered anomalous and excluded from the analysis if any of the following criteria were met: (1) pollutant concentrations exceeded the device’s upper detection limits; (2) values fell outside plausible indoor exposure ranges (e.g., PM_2.5_ > 500 μg/m^3^, PM_10_ > 600 μg/m^3^, CO_2_ > 5000 ppm, HCHO > 1.5 mg/m^3^, or TVOC > 5.0 mg/m^3^); or (3) all recorded values over the 24 h period were zero, indicating potential device malfunction. After excluding invalid or abnormal data (*n* = 16), a total of 219 participants were included in the final analysis (Grades 1–4: 66.2%; boys: 52.5%). Figure S1 presents the study’s screening flowchart. Daytime was defined as 8:00 to 17:00, corresponding to typical school hours when students primarily stayed in classrooms on weekdays. Nighttime was defined as 17:00 to 8:00 the following morning, when students predominantly stayed in their home bedrooms.

Multiple exposure metrics were calculated to comprehensively characterize indoor pollutant exposure. Specifically, the following exposure metrics were derived to capture variations in indoor pollutant levels across different locations and time periods: (1) Classroom 24 h exposure was determined by averaging pollutant concentrations in classrooms over each 24 h monitoring period, followed by computation of the mean across the seven-day measurement period. (2) Bedroom 24 h exposure was calculated using the same approach, averaging pollutant concentrations in bedrooms over each 24 h period and then across the entire week. (3) Overall exposure was computed as a time-weighted average of classroom and bedroom concentrations. For weekdays, daily exposure estimates incorporated classroom concentrations from 08:00 to 17:00 and bedroom concentrations from 17:00 to 08:00 the following morning. For weekends, exposure assessment was based exclusively on 24 h bedroom concentrations. The final weekly value represented the average of daily exposures across all seven days. (4) Location- and time-specific exposure metrics were also calculated, including the following: classroom exposure during weekday daytime (08:00–17:00); bedroom exposure during weekday daytime (08:00–17:00); bedroom exposure during weekday nighttime (17:00–08:00); bedroom exposure during weekend daytime (08:00–17:00); and bedroom exposure during weekend nighttime (17:00–08:00). For each metric, daily averages were first calculated within the specified period and then averaged over the seven-day monitoring duration.

### 2.3. Assessment of Depressive Symptoms

DS were assessed using the Chinese version of the Centre for Epidemiologic Studies Depression Scale (CES-D) [18,19], administered through a student questionnaire. This validated 20-item self-report instrument is widely used in population-based study to evaluate the frequency of each symptom experienced in the past week [18]. Each item is scored from 0 to 3, yielding a total score ranging from 0 to 60. The CES-D had good reliability and validity in the Chinese adolescent population (Cronbach’s α = 0.88) [20]. In our study, a score of ≥20 on the CES-D scale was defined as having depressive symptoms, as supported by validation studies suggesting improved specificity for Chinese adolescents [7,21,22]. Both the continuous CES-D score and the binary classification of clinically relevant DS were used as outcomes of interest in our analyses. A total of 130 participants provided complete CES-D data (Figure S1).

### 2.4. Covariates

Demographic, lifestyle, and household environmental information was collected through self-reported questionnaires completed by students and their parents. Demographic variables included age, grade, sex, and maternal education. Household environmental factors included recent home renovation (within the past year, yes/no), ventilation practices (primary ventilation method, window/machine) and regular use of air purifiers (yes/no). Lifestyle factors assessed at baseline included average daily sleep duration, number of days meeting recommended intake of fruits, vegetables, and protein; sugar-sweetened beverage (SSB) consumption; physical activity; and daily recreational screen time. Based on previous studies [23,24,25,26,27], five key unhealthy lifestyle factors associated with depressive symptoms were identified: (1) being in the sex- and age-specific lower half of physical activity distribution; (2) failing to meet fruit, vegetable, and protein intake recommendations in the past seven days; (3) consuming SSBs in the past seven days; (4) insufficient sleep (<10 h/day for primary school students, <9 h/day for secondary school students); and (5) recreational screen time exceeding 2 h/day. Participants were categorized into two lifestyle groups: favorable (0–2 unfavorable factors) and unfavorable (3–5 unfavorable factors). Additionally, we collected bullying experiences via student questionnaires. Details on these covariates are provided in the Supplemental Methods and Table S2.

### 2.5. Statistical Analysis

The distributions of classroom 24 h exposure, bedroom 24 h exposure, overall exposure, and exposures were described for specific location-time combinations, including classroom during weekday daytime (08:00–17:00), bedroom during weekday daytime (08:00–17:00), bedroom during weekday nighttime (17:00–08:00), bedroom during weekend daytime (08:00–17:00), and bedroom during weekend nighttime (17:00–08:00), using seven descriptive statistics: mean, standard deviation (SD), median, 25th percentile (P25), 75th percentile (P75), minimum, and maximum. In accordance with Indoor Air Quality Standard of China (GB/T 18883-2022) [15], exceedance rates were also calculated for each exposure metric, defined as the proportion of participants whose exposure levels surpassed the corresponding recommended limits.

Furthermore, the distributions of overall exposure and the associated exceedance rates were characterized across key subgroups using the same statistical indicators. These subgroups included sex (boys vs. girls), grade level (Grades 1–4 vs. Grade 7), maternal education level (bachelor’s degree or above vs. high school or below), recent home decoration within the past year (yes vs. no), and season (cool vs. warm). For each subgroup analysis, only participants with complete data for the corresponding stratification variable were included. Exceedance rates for each subgroup were similarly defined as the proportion of participants whose exposure levels exceeded the relevant standards.

Moreover, multivariable logistic regression models were used to assess the associations between overall exposure to indoor PM_2.5_, PM_10_, CO_2_, HCHO, and TVOC and the presence of depressive symptoms. In single-pollutant models, exposure to each air pollutant was separately included in the model to estimate the odds ratio (OR) of depressive symptoms and its 95% confidence interval (CI) per interquartile range (IQR) increase of exposure. The models were adjusted for a common set of confounders: age, sex, maternal education, number of unfavorable lifestyle factors, peer bullying, indoor temperature and relative humidity, recent home renovation (yes/no), primary ventilation method (window/mechanical), use of air purifiers (yes/no), and season at the time of indoor monitoring (warm/cold). To minimize data loss, missing values for categorical covariates were coded as an “unknown” category and retained in the models. Covariates included in the model were mutually adjusted.

In addition, we employed generalized linear regression models to examine the associations between overall exposure to the same pollutants and CES-D scores treated as a continuous outcome. These models were adjusted for the same set of covariates, and effect estimates represent the change in CES-D score per IQR increase in pollutant exposure. As a sensitivity analysis, we also explored an alternative threshold for defining depressive symptoms (CES-D ≥ 16) to assess the robustness of the findings. All statistical analyses were performed using R version 4.4.3 [28]. All statistical tests were 2-sided and *p* < 0.05 was considered statistically significant.

## 3. Results

### 3.1. Basic Characteristics of the Participants

A total of 219 children and adolescents were included in the final analysis, with a mean age of 9.9 years (SD = 2.1). Among them, 52.5% (*n* = 115) were boys and 47.5% (*n* = 104) were girls. Overall, 145 participants (66.2%) were in grades 1 to 4 of primary school, while 74 (33.8%) were in grade 7 of middle school. Regarding residential distribution, 27 participants (12.3%) resided in Dongcheng District, 121 (55.3%) in Tongzhou District, and 71 (32.4%) in Changping District. Based on the season during which indoor pollutants were measured, 121 participants (55.3%) were monitored during the warm season (April to October) and 98 (44.7%) during the cool season (November to March).

In terms of maternal education, 44.3% of mothers held a bachelor’s degree or higher, 34.2% had a high school education or below, and 21.5% of this information was missing. Additionally, 34.2% of participants reported that their homes had undergone decoration within the past year, 60.3% reported no recent decoration, and 5.5% did not provide this information. The basic characteristics of the study participants are summarized in Table 1.

### 3.2. Levels of Exposure to Seven Indoor Environmental Factors Cross Different Exposure Settings and Time Periods

Based on measured concentrations in classrooms and bedrooms, the median overall indoor exposure levels among children and adolescents in Beijing were 29.97 μg/m^3^ [19.02, 50.52] for PM_2.5_, 32.96 μg/m^3^ [14.87, 57.86] for PM_10_, and 965.87 ppm [699.10, 1295.92] for CO_2_. For gaseous pollutants, the median concentrations were 0.02 mg/m^3^ [0.01, 0.04] for HCHO and 0.32 mg/m^3^ [0.20, 0.47] for TVOC. The corresponding median values for indoor temperature and relative humidity were 25.10 °C [23.16, 27.42] and 39.39% [33.43, 47.57], respectively (Table 2).

Among the different exposure settings, classroom environments during weekday daytime exhibited the highest median concentrations of PM_2.5_ (29.70 μg/m^3^ [19.58, 47.16]), PM_10_ (36.28 μg/m^3^ [21.53, 51.74]), and TVOC (0.33 mg/m^3^ [0.21, 0.44]), while bedroom environments during weekend nighttime showed the highest median concentration of CO_2_ (1000.23 ppm [688.03, 1544.20]). The highest median concentrations of HCHO (0.02 mg/m^3^ [0.01, 0.03]), temperature (25.45 °C [23.29, 27.58]), and humidity (40.58% [34.79, 49.02]) were observed in bedrooms during weekday nighttime (Table S3).

**Figure 1:**
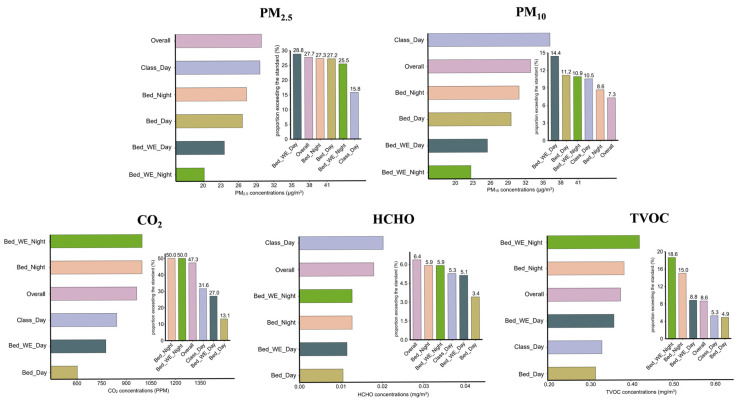
Median concentrations and exceedance rates of five indoor air pollutants across different exposure settings and time periods. Horizontal bar charts represent the median concentrations of five indoor air pollutants (PM_2.5_, PM_10_, CO_2_, HCHO, and TVOC) across various indoor exposure settings and time periods, including weekday/weekend and classroom/bedroom contexts. Vertical bar charts show the corresponding proportions of samples exceeding the recommended limits defined in the Indoor Air Quality Standard (GB/T 18883-2022) [15] of China (see Supplementary Table S1 for exact threshold values). Abbreviations: PM_2.5_, fine particulate matter; PM_10_, inhalable particulate matter; CO_2_, carbon dioxide; HCHO, formaldehyde; TVOC, total volatile organic compounds. Class_Day, classroom during weekday daytime. Bed_Day, bedroom during weekday daytime. Bed_Night, bedroom during weekday nighttime. Bed_WE_Day, bedroom during weekend daytime. Bed_WE_Night, bedroom during weekend nighttime. “Overall” represents the average across all time periods.

Regarding exceedance rates, bedrooms during weekend nighttime exhibited the highest rates for CO_2_, TVOC, and relative humidity, as shown in Figure 1 and Figure S3. The highest exceedance rates for PM_2.5_, PM_10_, and HCHO were observed in classrooms during weekday daytime (Figure 1), while temperature exceedances occurred most frequently in bedrooms during weekend daytime.

### 3.3. Distribution of Types of Exceedances Among Seven Indoor Environmental Indicators

**Figure 2:**
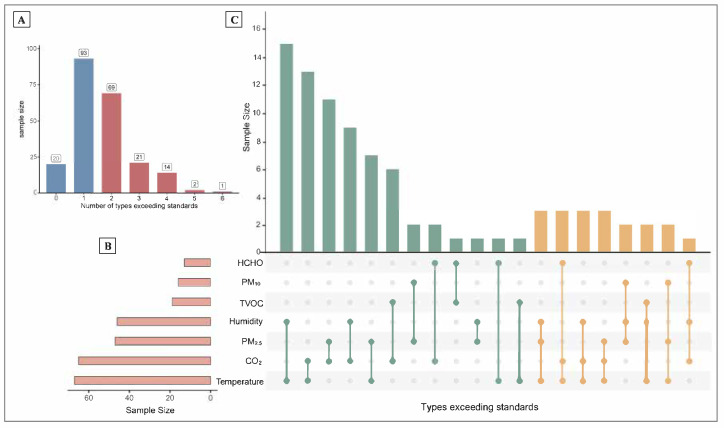
Distribution of types of exceedances among seven indoor environmental indicators. The top left bar chart (**A**) shows the number of samples exceeding 0 to 6 types of standards. The bottom left horizontal bar chart (**B**) represents the number of samples exceeding the standard for each individual indicator. The main bar chart on the right (**C**) displays the combinations of indicators that simultaneously exceeded the standards and their corresponding sample sizes. Each vertical bar indicates a specific combination of pollutants exceeding thresholds, as denoted by the connected filled dots below. The height of each bar reflects the number of samples with that particular combination of exceedances. Notably, the UpSet plot focuses on combinations involving simultaneous exceedances of two or three pollutants.

Based on Figure 2, among the seven indoor environmental indicators assessed, most participants exceeded one (*n* = 93) or two (*n* = 69) indicator standards. Temperature and CO_2_ were the indicators most frequently exceeding the recommended limits. The UpSet plot illustrates that combinations involving temperature, CO_2_, and PM_2.5_ were the most common types of simultaneous exceedances. Threshold values were defined based on China’s Indoor Air Quality Standard (GB/T 18883–2022) [15].

### 3.4. Levels of Exposure to Seven Indoor Environmental Factors Across Different Subgroups

**Figure 3:**
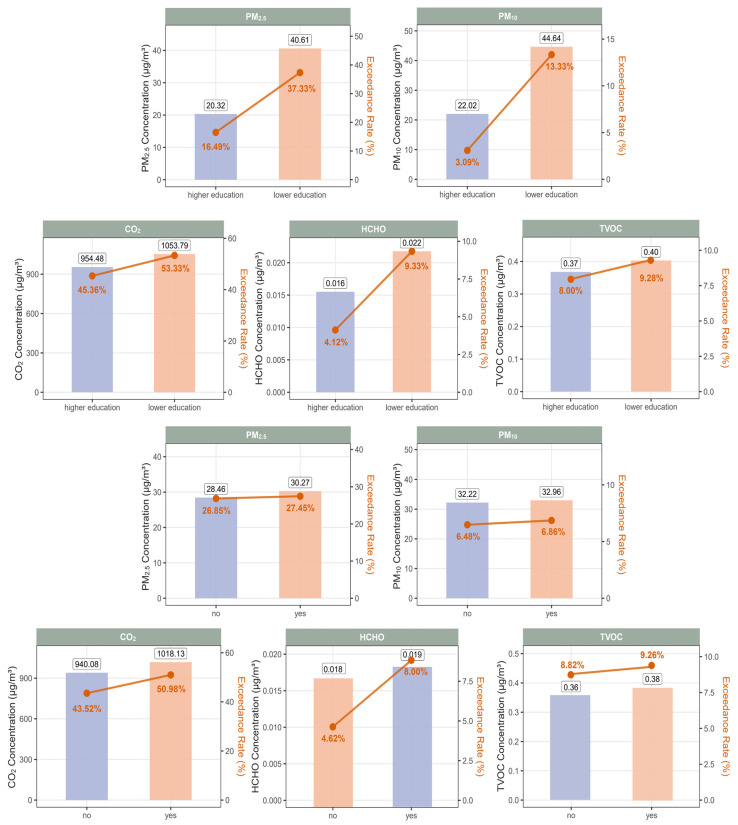
Median levels and exceedance rates of five indoor air pollutants by maternal education and recent home decoration within the past year. Bar heights represented the median concentrations of each pollutant in different subgroups, while the dots indicated the corresponding exceedance rates. Higher education indicated bachelor’s degree or above, and lower education indicated high school or below.

As shown in Figure 3 and Tables S4–S8, subgroup differences in median concentrations and exceedance rates varied across sociodemographic and household characteristics. In terms of sex and grade level (first and second rows), no consistent trends emerged across pollutants. Although girls exhibited slightly higher median concentrations and exceedance rates of CO_2_, HCHO, and TVOC, whereas boys had marginally higher PM_2.5_ and PM_10_ levels, none of these differences reached statistical significance (all *p* > 0.05; Table S4). Given the small effect sizes and the lack of adjustment for activity patterns or product usage, these trends should be interpreted cautiously. Similarly, students in Grade 7 showed higher median concentrations and exceedance rates of PM_10_ and HCHO, but lower levels of PM_2.5_, CO_2_, and TVOC compared to those in Grades 1–4; however, none of these differences reached statistical significance (all *p* > 0.05; Table S5).

In contrast, clear and consistent patterns were observed for maternal education level and recent home decoration (third and fourth rows). Children whose mothers had a lower level of education were exposed to higher median concentrations of all five pollutants and exhibited notably higher exceedance rates, particularly for PM_10_ (13.33% vs. 3.09%) and HCHO (9.33% vs. 4.12%) (Figure 3 and Table S6). Similarly, participants living in homes that had been renovated within the past year had higher median exposure levels and slightly elevated exceedance rates across all pollutants compared to those from non-renovated homes (Figure 3 and Table S7). In addition, seasonal differences were evident, with markedly higher CO_2_ concentrations and exceedance rates observed during the cool season compared to the warm season (median: 1169.14 vs. 552.95 PPM; exceedance rate: 72.45% vs. 15.49%) (Table S8).

### 3.5. Association of Exposure to Five Indoor Air Pollutants with Depressive Symptoms

Among the 130 participants with complete mental health questionnaire data, 34 (26.2%) were identified as having depressive symptoms. Of these, 58.8% were girls and 41.2% were boys; however, this gender difference was not statistically significant. In terms of grade distribution, 64.7% of children with depressive symptoms were in Grade 7, while 35.3% were in Grades 3–4 (Table S9).

**Figure 4:**
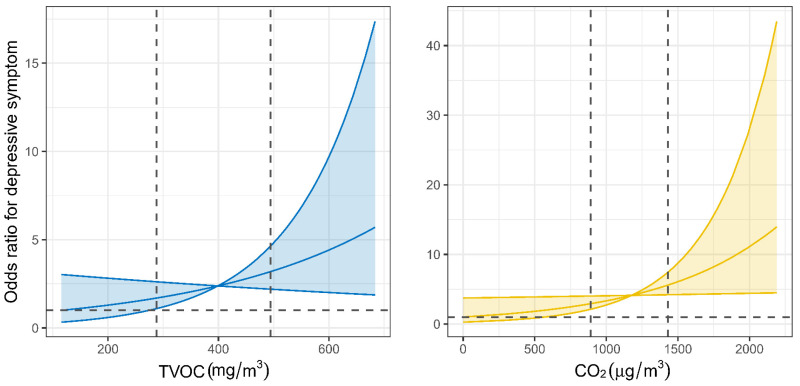
Exposure–response curves of exposure to indoor TVOC and CO_2_ with depressive symptoms. Indoor exposure was calculated as a time-weighted average of classroom and bedroom concentrations. For weekdays, daily exposure was estimated by averaging classroom concentrations from 08:00 to 17:00 and bedroom concentrations from 17:00 to 8:00 the next morning. For weekends, daily exposure was estimated using 24 h bedroom concentrations. The final combined exposure was obtained by averaging the daily values across the entire week. The solid lines with shaded regions represent odds ratio of depressive symptoms and their corresponding 95% CIs. The black horizontal line in each panel indicates the referent odds ratio of 1. The black horizontal line in each panel indicates the referent odds ratio of 1. The two vertical dashed lines indicate the 25th and 75th percentiles of exposure levels. CO_2_, carbon dioxide; TVOC, total volatile organic compounds.

In single-pollutant models, we observed significant associations between overall exposure to indoor CO_2_ and TVOC and the odds of depressive symptoms among children and adolescents. Specifically, each IQR increase in CO_2_ (552.38 PPM) and TVOC (0.21 mg/m^3^) exposure was associated with a 2.10-fold (95% CI: 1.07, 4.10; *p* = 0.03) and 1.83-fold (95% CI: 1.01, 3.35; *p* = 0.04) increase in the odds of depressive symptoms, respectively (Table 3). No significant associations were observed for PM_2.5_, PM_10_, or HCHO exposure. The exposure–response curves demonstrated an overall increasing trend in the odds of depressive symptoms with higher levels of TVOC and CO_2_ exposure. The ORs remained relatively stable or negligible at lower concentrations but began to rise more sharply beyond the 75th percentile of exposure for both pollutants (Figure 4). Consistent findings were observed when using CES-D scores as continuous outcomes, with higher exposures to CO_2_ and TVOC significantly associated with increased CES-D scores (Table S10). Similarly, sensitivity analyses using an alternative CES-D cutoff of ≥16 yielded comparable associations (Table S11), reinforcing the robustness of the main findings.

## 4. Discussion

This study employed portable monitoring devices to assess indoor exposure to environmental pollutants among 219 children and adolescents in Beijing, focusing on different microenvironments and time periods, and further explored their potential mental health implications. The results revealed clear spatial and temporal variations in pollutant levels. PM_2.5_, PM_10_, and TVOC concentrations were highest in classrooms during weekday daytime, whereas CO_2_ levels peaked in bedrooms on weekend nights. HCHO, temperature, and humidity were highest in bedrooms during weekday nights. Exceedance patterns corresponded to these peaks, with temperature, CO_2_, and PM_2.5_ most frequently exceeding standard limits. Most participants exceeded one or two pollutant indicators, with exceedances often occurring in combination. Children whose mothers had lower educational attainment or whose homes had undergone recent renovation experienced higher median concentrations and exceedance rates across all pollutants. In single-pollutant models, each IQR increase in CO_2_ and TVOC was associated with nearly a twofold increase in the odds of depressive symptoms. The exposure-response curves remained relatively flat at lower concentrations but exhibited a steep increase in the odds of depressive symptoms at higher exposure levels for both pollutants.

The observed spatiotemporal heterogeneity in indoor pollutant concentrations underscores the influence of varying microenvironmental contexts and time periods. Notably, the highest levels of PM_2.5_ and PM_10_ were observed in classrooms during weekday daytime, likely due to active student movement, resuspension of dust, chalk use, limited ventilation between classes, and infiltration of outdoor pollutants [29]. In contrast, the peak CO_2_ and TVOC concentrations occurred during weekend nighttime in bedrooms, reflecting prolonged enclosure during sleep, increased household occupancy, continuous emission of VOCs from building materials and reduced ventilation—particularly in winter [30]. This is further supported by our subgroup analysis (Table S8), which showed significantly higher median CO_2_ concentrations during the cool season compared to the warm season. These seasonal differences highlight the importance of improving ventilation practices during colder months to mitigate indoor air quality deterioration.

Elevated nighttime concentrations of HCHO, temperature, and humidity in bedrooms may be attributed to continuous emissions of volatiles from building materials and furniture [31], reduced nighttime air exchange, and human metabolic activity contributing to increased humidity [32]. Exceedance patterns indicated that temperature, CO_2_, and PM_2.5_ were the most frequently surpassed indicators, either alone or in combination, suggesting that thermal discomfort, inadequate ventilation, and particulate pollution are the primary indoor environmental challenges faced by children in urban Beijing. These findings align with previous research identifying CO_2_ as a proxy for ventilation adequacy [33] and highlight the difficulty of meeting multiple IAQ standards, particularly given regional climatic conditions and household behavioral practices.

Our findings identified significant sociodemographic disparities in indoor pollutant exposure, notably associated with maternal educational attainment and recent home renovation activities. Children whose mothers possessed lower levels of education consistently experienced higher median concentrations and exceedance rates of all assessed pollutants. This pattern likely reflects underlying socioeconomic inequalities, as lower maternal education often correlates with reduced household income and limited access to superior housing conditions [34,35], characterized by inadequate ventilation, smaller living spaces, proximity to outdoor pollution sources, and the use of low-cost, high-emission materials. Additionally, disparities in environmental health literacy and behaviors, such as ventilation practices, cleaning routines, and renovation choices, may further exacerbate exposure risks [36]. Constraints in financial and informational resources may further hinder households’ ability to adopt air purification technologies and implement other environmental enhancements.

Similarly, recent home renovation was characterized by elevated pollutant levels and exceedance rates, highlighting the role of construction materials, adhesives, coatings, and new furniture as major sources of indoor pollutants [37], particularly VOCs like TVOC and HCHO. These emissions may persist long after renovation, leading to prolonged exposure among children. Consistent with previous research, our results underscore how socioeconomic status and residential conditions shape indoor environmental quality and contribute to environmental health disparities. These findings carry significant public health implications, indicating the need for targeted interventions such as public education campaigns, post-renovation air quality assessments, and financial support for improving ventilation in disadvantaged households.

Our supplementary analyses further demonstrated that geographic location significantly influenced indoor exposure to PM through outdoor infiltration. We used high-spatial-resolution (1 km × 1 km) PM_2.5_ and PM_10_ data from the China High Air Pollutant (CHAP) dataset, extracting daily concentrations at each participant’s residential and school locations during the monitoring period and calculating 7-day average outdoor exposure levels. We observed strong positive correlations between indoor and outdoor concentrations (Spearman’s r = 0.62 for PM_2.5_, 0.68 for PM_10_; both *p* < 0.001), confirming outdoor air infiltration as a critical determinant of indoor PM levels. Stratified analyses revealed that this relationship was particularly pronounced during high-pollution episodes (defined as days with outdoor PM_2.5_ > 75 μg/m^3^), with indoor–outdoor correlations increasing to r = 0.82 (*p* < 0.001). These results suggest substantial penetration of outdoor PM into indoor environments during haze or pollution-heavy periods. This finding aligns with prior evidence [38], which demonstrated that rigorously calibrated low-cost sensors can effectively capture indoor PM_2.5_ fluctuations under haze conditions in Beijing. In contrast, spatiotemporal patterns suggested minimal geographic influence on TVOC, CO_2_, and HCHO, as these pollutants appeared dominated by microenvironment-specific indoor sources such as classroom activities, occupancy patterns, ventilation practices, and building materials.

The observed associations between elevated indoor CO_2_ and TVOC levels and increased odds of depressive symptoms in children are supported by plausible biological mechanisms. Even at sub-toxic levels, CO_2_ may disrupt neurophysiological homeostasis by altering cerebral blood flow, influencing blood pH regulation, and interfering with neurotransmitter systems such as serotonin and gamma-aminobutyric acid, which are both essential for emotional stability [39,40]. Similarly, TVOCs, which include compounds such as benzene, toluene, and formaldehyde, are known to possess neurotoxic and endocrine-disrupting properties. These substances may affect brain function by interacting directly with limbic structures, initiating systemic inflammation and oxidative stress, or disrupting the hypothalamic–pituitary–adrenal axis [14,41,42], all of which have been implicated in the development of depressive symptoms. In addition, exposure to TVOCs may impair sleep quality through mucosal irritation or unpleasant odors, both of which are closely linked to emotional disturbances. Beyond its role as a specific gas, CO_2_ should be regarded as a comprehensive indicator of overall indoor environmental quality. Elevated CO_2_ levels commonly reflect insufficient ventilation, which facilitates the accumulation of various indoor pollutants including TVOCs, metabolic byproducts, and bioaerosols [43]. Prolonged exposure to this poorly ventilated and multi-pollutant environment may contribute to chronic stress on the developing nervous system, thereby increasing the risk of adverse mental health outcomes in children.

Beyond pollutant-specific effects, it is essential to account for the potential influence of other psychosocial and environmental stressors that may confound or interact with the impacts of indoor air pollution. For instance, parental mental health conditions can affect children’s emotional well-being both directly and indirectly by shaping parenting practices and the management of the home environment. Likewise, indoor noise originating from neighbors or nearby traffic, suboptimal sleep conditions, and co-exposure to additional stressors such as household crowding, light pollution, or exacerbated thermal discomfort may independently contribute to depressive symptoms or amplify the effects of indoor pollutants. These unmeasured variables may partially explain the observed associations and highlight the importance of future studies to incorporate the observed associations and highlight the need for research incorporating more comprehensive assessments of psychosocial and environmental exposures.

The exposure–response curves for CO_2_ and TVOC demonstrated a distinct non-linear pattern, characterized by relatively stable associations at lower concentrations and sharply increased risks beyond the 75th percentile (approximately 1295 PPM for CO_2_ and 0.47 mg/m^3^ for TVOC). This threshold-like effect suggests the existence of biological tipping points, beyond which homeostatic mechanisms may fail and neurotoxic processes become more easily activated. These findings underscore the need for targeted interventions, as maintaining indoor pollutant levels below these thresholds may substantially reduce the risk of depressive symptoms in children. Furthermore, comparison with current regulatory standards (e.g., GB/T 18883-2022) [15] suggests that environments compliant with existing limits may still pose mental health risks if thresholds relevant to neuropsychological outcomes are exceeded. The similar response patterns observed for CO_2_ and TVOC highlight the importance of integrated strategies that both minimize indoor emission sources and ensure sufficient ventilation. Such approaches are particularly vital for safeguarding the mental health of children in densely populated urban environments.

This study provides novel insights into the mental health implications of indoor air pollution among children and adolescents. To our knowledge, it is among the first to identify associations between indoor CO_2_ and TVOC exposures and increased odds of depressive symptoms in Chinese children and adolescents, highlighting inadequate ventilation and VOC contamination as critical environmental risk factors. These findings hold substantial public health significance, given the widespread exceedances of pollutant levels observed in classrooms during the day and bedrooms at night, as well as the disparities linked to socioeconomic factors such as low maternal education and recent home renovations.

The results offer practical implications for both institutional and household environments. In school settings, improving classroom ventilation, especially during teaching hours, and minimizing chalk dust or cleaning-related particulate emissions are critical. In households, interventions such as selecting certified low-emission renovation materials, ensuring adequate airing-out periods following home decoration, and adopting healthy ventilation practices are strongly recommended. Moreover, our findings underscore the need for scalable and evidence-based strategies to reduce pollutant exposure. These may include the use of HEPA-equipped air purifiers to effectively reduce PM concentrations, activated carbon filters to remove VOCs and formaldehyde, and optimized window ventilation during periods of lower outdoor pollution to mitigate CO_2_ buildup. Behavioral modifications—such as limiting the use of incense or high-emission cleaning products and avoiding overcrowding—may also contribute to a healthier indoor air environment. Taken together, these interventions can inform school policies, parental practices, and environmental health guidelines to better protect child and adolescent mental health in densely populated urban settings.

Several limitations should be acknowledged. First, the cross-sectional design of this study limits any causal interpretation. While we observed significant associations between indoor pollutant exposures and depressive symptoms, these findings reflect correlations rather than causal relationships. It is also possible that children experiencing depressive symptoms may alter their behavior or environmental interactions (e.g., reduced ventilation), leading to reverse causality. Therefore, the observed associations should be interpreted with caution. Second, exposure assessments were based on short-term measurements in specific indoor settings, which may not capture continuous or individual-level exposures. Important pollutants such as nitrogen dioxide, ozone, and specific VOC components were also not included. Third, depressive symptoms were evaluated using self-reported scales rather than clinical diagnoses, which may lead to misclassification or reporting bias. Fourth, although our models adjusted for several covariates, residual confounding cannot be ruled out. Unmeasured factors such as parental mental health, family dynamics, indoor noise, and other psychosocial or environmental stressors may independently affect children’s mental health or interact with indoor air pollutants, potentially influencing the observed associations. In addition, detailed parental tobacco-use data were collected but excluded from primary analyses due to high missingness (see Supplemental Questionnaire, Q1–Q4), raising concerns about selection bias. Moreover, we did not specifically assess episodic indoor pollution sources (e.g., candles or incense) or cooking fuel types (e.g., gas stoves). Nevertheless, given the episodic nature of candle/incense use and the high prevalence of gas stove use (>95%) in Beijing, confounding from these sources is likely limited. Finally, although we collected information on home renovation and the primary type of ventilation (i.e., window-based vs. mechanical), we did not systematically assess seasonal variations in ventilation frequency or human occupancy patterns, including room usage or number of occupants. The absence of these data may have introduced residual confounding in the analysis. Fifth, while a higher proportion of depressive symptoms was observed among girls in our sample, this difference did not reach statistical significance due to the limited number of affected participants. This trend should be interpreted with caution but may indicate potential gender differences in vulnerability to indoor environmental exposures, warranting further investigation in larger, gender-stratified studies. Finally, the study sample was drawn from three districts in Beijing using convenience sampling, which limits the generalizability of the findings. As participants were recruited from the natural control group of a school-based intervention study, they may not fully represent the broader urban child population. Families with higher parental education or greater health awareness may have been more likely to participate, introducing potential selection bias. While the inclusion of districts with varying levels of urbanization adds some contextual diversity, caution is warranted when extrapolating absolute exposure levels or subgroup distributions to other populations. Future studies should adopt longitudinal designs; apply continuous and personalized exposure monitoring; investigate specific pollutants, activity diaries, and biological pathways; and assess the effectiveness of air quality interventions in broader and more diverse populations.

## 5. Conclusions

Overall, this study revealed that children and adolescents in Beijing were exposed to varying levels of indoor air pollutants across different microenvironments and time periods, with some concentrations exceeding recommended thresholds. Higher levels of PM and TVOC were observed in classrooms during the daytime, while CO_2_ concentrations tended to peak in bedrooms at night. Exposure disparities were more pronounced among children from households with lower maternal education levels and those living in recently renovated homes. It was found that elevated indoor CO_2_ and TVOC levels were significantly associated with increased odds of depressive symptoms among children and adolescents. These findings provide timely and important evidence to support targeted environmental interventions and public health strategies. They may inform the development of school-based air quality policies and urban building codes aimed at improving ventilation and reducing indoor pollutant sources to safeguard the mental well-being of children and adolescents, particularly in densely populated cities in China. Further longitudinal studies are warranted to investigate the long-term impacts of indoor air pollution on the mental health of children and adolescents.

## Figures and Tables

**Table 1:** Characteristics of 219 study participants.

Characteristics	Overall (N = 219)
Age, mean ± SD	9.9 ± 2.1
Sex, *n* (%)	
Boys	115 (52.5%)
Girls	104 (47.5%)
Grade, *n* (%)	
Grade 1	9 (4.1%)
Grade 2	38 (17.4%)
Grade 3	45 (20.5%)
Grade 4	53 (24.2%)
Grade 7	74 (33.8%)
District, *n* (%)	
Dongcheng	27 (12.3%)
Tongzhou	121 (55.3%)
Changping	71 (32.4%)
Season ^a^, *n* (%)	
Warm	121 (55.3%)
Cool	98 (44.7%)
Maternal education, *n* (%)	
Bachelor’s degree or above	97 (44.3%)
High school or below	75 (34.2%)
Unknown	47 (21.5%)
Home decoration within one year, *n* (%)	
Yes	75 (34.2%)
No	132 (60.3%)
Unknown	12 (5.5%)

^a^ Cool season was defined as November to March, while warm season was defined as April to October, corresponding to the seasons during which indoor pollutants were measured using portable monitoring devices.

**Table 2:** Distributions of seven indoor environmental factors among 219 children and adolescents in Beijing, based on 7-day averaged 24 h exposures in classrooms and bedrooms.

Exposure	Units	Mean ± SD	Median (Q_25_–Q_75_)	Min	Max
Classroom ^a^					
PM_2.5_	μg/m^3^	41.82 ± 33.37	35.55 (22.05–53.36)	2.23	131.13
PM_10_	μg/m^3^	46.32 ± 36.79	39.24 (24.67–57.79)	2.7	145.12
CO_2_	PPM	644.77 ± 364.05	595.30 (549.09–741.57)	10.11	1819.3
HCHO	mg/m^3^	0.02 ± 0.01	0.01 (0.01–0.02)	0.01	0.04
TVOC	mg/m^3^	0.31 ± 0.16	0.30 (0.20–0.44)	0.10	0.58
Temperature	°C	23.01 ± 5.43	24.03 (21.18–25.54)	11.84	33.92
Humidity	%	32.94 ± 9.50	32.66 (25.01–39.76)	19.48	49.85
Bedroom ^b^					
PM_2.5_	μg/m^3^	40.10 ± 49.61	27.89 (12.19–50.78)	0.47	494.26
PM_10_	μg/m^3^	44.40 ± 56.52	31.42 (14.07–55.35)	0.71	589.18
CO_2_	PPM	1031.51 ± 527.11	919.21 (667.95–1293.89)	0.00	3109.33
HCHO	mg/m^3^	0.03 ± 0.07	0.01 (0.01–0.03)	0.00	0.87
TVOC	mg/m^3^	0.39 ± 0.16	0.39 (0.28–0.48)	0.10	0.94
Temperature	°C	25.51 ± 3.13	25.45 (23.19–27.63)	15.5	33.52
Humidity	%	40.07 ± 9.48	39.07 (33.66–47.32)	18.55	64.19
Overall ^c^					
PM_2.5_	μg/m^3^	41.36 ± 48.85	29.97 (12.90–52.52)	0.59	383.21
PM_10_	μg/m^3^	45.90 ± 55.42	32.96 (14.87–57.86)	0.79	453.3
CO_2_	PPM	1037.86 ± 490.70	965.87 (699.60–1295.92)	0.00	3025.33
HCHO	mg/m^3^	0.03 ± 0.08	0.02 (0.01–0.03)	0.00	0.77
TVOC	mg/m^3^	0.39 ± 0.14	0.38 (0.29–0.47)	0.12	1.09
Temperature	°C	25.34 ± 3.20	25.10 (23.16–27.42)	14.99	33.99
Humidity	%	39.84 ± 8.93	39.39 (33.43–47.57)	18.63	57.59

SD, standard deviation; Q_25_, the 25th percentile; Q_75_, the 75th percentile; Min, Minimum; Max, Maximum; PM_2.5_, particulate matter with an aerodynamic diameter of ≤2.5 μm; PM_10_, particulate matter with an aerodynamic diameter of ≤10 μm; CO_2_, carbon dioxide; HCHO, formaldehyde; TVOC, total volatile organic compounds. ^a^ Classroom 24 h exposure was determined by averaging classroom concentrations over each 24 h monitoring period, followed by averaging across the entire 7-day measurement period. ^b^ Bedroom 24 h exposure was similarly computed by averaging bedroom concentrations over each 24 h monitoring period and then across the full week. ^c^ Overall exposure was calculated as a time-weighted average of classroom and bedroom concentrations. For weekdays, daily exposure was estimated by averaging classroom concentrations from 08:00 to 17:00 and bedroom concentrations from 17:00 to 8:00 the next morning. For weekends, daily exposure was estimated using 24 h bedroom concentrations. The final combined exposure was obtained by averaging the daily values across the entire week.

**Table 3:** Associations between exposure to indoor air pollutants and depressive symptoms among children and adolescents.

Exposure ^a^	Increment (IQR)	ORs (95% CIs) ^b^	*p* Values
PM_2.5_	39.44 μg/m^3^	1.08 (0.73, 1.58)	0.70
PM_10_	43.83 μg/m^3^	1.08 (0.75, 1.57)	0.67
CO_2_	552.38 PPM	2.10 (1.07, 4.10)	0.03 *
HCHO	0.02 mg/m^3^	1.12 (0.79, 1.58)	0.17
TVOC	0.21 mg/m^3^	1.83 (1.01, 3.35)	0.04 *

PM_2.5_, particulate matter with an aerodynamic diameter of ≤2.5 μm; PM_10_, particulate matter with an aerodynamic diameter of ≤10 μm; CO_2_, carbon dioxide; HCHO, formaldehyde; TVOC, total volatile organic compounds. OR, odds ratio; CI, confidence interval. ^a^ Indoor exposure was calculated as a time-weighted average of classroom and bedroom concentrations. For weekdays, daily exposure was estimated by averaging classroom concentrations from 08:00 to 17:00 and bedroom concentrations from 17:00 to 8:00 the next morning. For weekends, daily exposure was estimated using 24 h bedroom concentrations. The final combined exposure was obtained by averaging the daily values across the entire week. ^b^ Effect estimates were odds ratios of depressive symptoms associated with an interquartile increase in indoor environmental exposure. Associations were adjusted for a common set of confounders: age, sex, maternal education, number of unfavorable lifestyle factors, peer bullying, indoor temperature and relative humidity, recent home renovation, primary ventilation method, use of air purifiers, and season at the time of indoor monitoring. Factors listed in the confounder set were adjusted for each other. * Two-tail significance level was set at *p* < 0.05.

## Data Availability

Due to ethical considerations and confidentiality agreements, the data used in this study are not publicly available.

## Supplementary Materials

The following supporting information can be downloaded at https://www.mdpi.com/article/10.3390/toxics13070563/s1. Figure S1: Flowchart of participants’ inclusion; Figure S2: Spearman correlation coefficients between indoor environmental exposures based on weighted average concentrations; Figure S3: Median levels and exceedance rates of two indoor environmental parameters across different exposure settings and time periods; Table S1: Parameters of the portable indoor air pollution monitoring device; Table S2: Covariates and data processing methods; Table S3: Distributions of 7 indoor environmental factors among 219 children and adolescents in Beijing across different exposure settings and time periods; Table S4: Distributions of time-weighted overall exposure to seven indoor environmental factors among 219 children and adolescents in Beijing, by sex; Table S5: Distributions of time-weighted overall exposure to seven indoor environmental factors among 219 children and adolescents in Beijing, by grade; Table S6: Distributions of time-weighted overall exposure to seven indoor environmental factors among 219 children and adolescents in Beijing, by maternal education; Table S7: Distributions of time-weighted overall exposure to seven indoor environmental factors among 219 children and adolescents in Beijing, by decoration; Table S8: Distributions of time-weighted overall exposure to seven indoor environmental factors among 219 children and adolescents in Beijing, by season; Table S9: Characteristics of 130 study participants with and without depressive symptoms; Table S10: Associations between exposure to indoor air pollutants and CES-D scores among children and adolescents; Table S11: Associations between exposure to indoor air pollutants and depressive symptoms (using CES-D scores ≥ 16) among children and adolescents.
