# Supplementary material for: Spatiotemporal Patterns of Indoor Air Pollution and Its Association with Depressive Symptoms Among Schoolchildren in China
Source: Toxics. 2025 Jul 1;13(7):563. doi: 10.3390/toxics13070563 (PMC12299042; doi:10.3390/toxics13070563)
Supplement: Supplementary file 1 [file toxics-13-00563-s001.zip › toxics-3705041-supplementary.pdf]

Supplemental Methods

Assessment of Covariates

Physical activity

Physical activity was assessed using the International Physical Activity Questionnaire-Short

occupational, commuting, domestic, and leisure-time activities in the past 12 months. Total physical activity levels (metabolic equivalent task-hours per day, MET-h/d) were calculated by multiplying hours spent on each activity per day by corresponding physical intensity and then summing up the MET-h/d for all activities.

Sleep

Furthermore, the questionnaire utilized for gathering information on sleep duration was adapted from the Pittsburgh Sleep Quality Index. Sleep duration data were collected independently for weekdays and weekends, and the average daily sleep duration was determined using the formula: Average daily sleep duration = (weekday sleep duration ×5 + weekend sleep duration × 2) / 7. In accordance with the stipulated daily sleep duration requirements outlined in the Healthy China Action Plan (2019–2030), insufficient sleep was categorized as <10 h per day for primary school students, <9 h per day for junior high school students, <8 h per day for high school students, and <7 h per day for college students or adults.

Sugar-sweetened beverages (SSBs)

Finally, the assessment of sugary beverage consumption was based on the days consumed SSBs in the past 7 days. To align with the Chinese Dietary Guidelines and the specific attributes of the study population, we categorized SSBs consumption as no consumption or 1-7 days.

Recreational screen time

Recreational screen time refers to the daily duration of leisure activities involving electronic screens, including watching television, playing video games, or using social media for entertainment. In line with the Implementation Program for Comprehensive Prevention and Control of Nearsightedness in Children and Adolescents, recreational screen time was categorized into two groups: individuals with less than 2 hours per day and those with 2 or more hours per day.

Days met fruit/vegetable and protein intake

Fruit and vegetable intake, as well as protein consumption, were assessed based on the number of days participants consumed at least five servings of fruits and vegetables and one serving of high-quality protein over the past seven days. This evaluation aligns with the WHO recommendations for a healthy diet (https://www.who.int/zh/news-room/fact-sheets/detail/healthy-diet). Intake was categorized into two groups: "Did not meet intake requirements" and "Met intake requirements (1–7 days)".

Table S1. Parameters of the portable indoor air pollution monitoring device.

Exposure	Units	Resolution	Measurement range	Accuracy deviation	Standard limit
$PM_{2.5}$	μg/m^3	1 μg/m^3	0-1000 μg/m^3	±10% of the number or ± 10 μg/m^3	⩽0.05 mg/m^3
PM_{10}	μg/m^3	1 μg/m^3	0-1000 μg/m^3	±10% of the number or ± 10 μg/m^3	⩽0.10 mg/m^3
CO_2	PPM	1 PPM	0-5000 PPM	<±10% of the number or ±70 PPM	⩽0.10%
HCHO	mg/m^3	0.01 mg/m^3	0-2 mg/m^3	±20% of the number or ± 0.02 mg/m^3	⩽0.08 mg/m^3
TVOC	mg/m^3	0.01 mg/m^3	0-3 mg/m^3	±20% of the number or ± 0.06 mg/m^3	⩽0.60 mg/m^3
Temperature	℃	0.1 ℃	0-60 ℃	0.5 ℃	22-28℃ in warm season;16-24℃ in cool season[a]
Humidity	%	1%	0-99%	10%	40-80% in warm season; 30-60% in cool season[a]

$PM_{2.5}$, particulate matter with an aerodynamic diameter of ≤2.5 μm; PM_{10}, particulate matter with an aerodynamic diameter of ≤10 μm; CO_2, carbon dioxide; HCHO, formaldehyde; TVOC, total volatile organic compounds.

[a]Cool season was defined as November to March, while warm season was defined as April to October.

Table S2. Covariates and data processing methods.

Field	Item	Processing	Coding	Coding notes
**Demographics**	Grade	Categorical	4th grade	Set as 0
			7th grade	Set as 1
			NA	Set as NA
	Sex	Categorical	Male	Set as 0
			Female	Set as 1
			NA	Set as NA
	Maternal Education	Categorical	Bachelor's degree or above	Set as 0
			High school or below	Set as 1
			NA	Set as NA
**Lifestyle factors**	Weekdays sleep duration	Continuous	/	Calculate the difference between the Wake-up Time and Bedtime.
			NA	Set as NA
	Weekends sleep duration	Continuous	/	Calculate the difference between the Wake-up Time and Bedtime.
			NA	Set as NA
	Sleep duration	Categorical	Sufficient (≥10 h per day for primary school students, ≥9 h per day for junior high school students)	Set as 0
			Insufficient (<10 h per day for primary school students, <9 h per day for junior high school students)	Set as 1
			NA	Set as NA
	Days met fruit/vegetable and protein intake	Categorical	0	Set as 1, Did not meet intake
			1	Set as 0, Met intake (1–7 days)
			2	Set as 0, Met intake (1–7 days)

			3	Set as 0, Met intake (1–7 days)
			4	Set as 0, Met intake (1–7 days)
			5	Set as 0, Met intake (1–7 days)
			6	Set as 0, Met intake (1–7 days)
			7	Set as 0, Met intake (1–7 days)
			NA	Set as NA
	Days consumed SSBs (past 7 days)	Categorical	0	Set as 0, No consumption
			1	Set as 1, 1–7 days
			2	Set as 1, 1–7 days
			3	Set as 1, 1–7 days
			4	Set as 1, 1–7 days
			5	Set as 1, 1–7 days
			6	Set as 1, 1–7 days
			7	Set as 1, 1–7 days
			NA	Set as NA
	Physical activity	Categorical	Low level (MET ≤ sex- and age-specific median cutoffs)	Set as 1
			High level (MET ≥ sex- and age-specific median cutoffs)	Set as 0
			NA	Set as NA
	Recreational screen time	Categorical	<2 h per day (based on average daily duration of Recreational screen time)	Set as 0
			⩾2 h per day (based on average daily duration of Recreational screen time)	Set as 1
			NA	Set as NA

**Peer-Related emotional distress**	Bullied by peers this semester	Categorical	Yes	Set as 1
			No	Set as 0
			NA	Set as NA
**Season at the time of indoor monitoring**	Date of indoor monitoring	Categorical	Warm	Set as 0
			Cold	Set as 1
**Recent home renovation**	Home renovation within one year	Categorical	Yes	Set as 1
			No	Set as 0
**Primary ventilation method**	Regular ventilation method	Categorical	Window	Set as 1
			Mechanical	Set as 0
**Regular use of air purifiers**	Regular use of air purifiers	Categorical	Yes	Set as 1
			No	Set as 0

Table S3. Distributions of 7 indoor environmental factors among 219 children and adolescents in Beijing across different exposure settings and time periods[a].

Exposure	Units	Mean± SD	Median (Q_{25}-Q_{75})	Min	Max
**Classroom during weekdays daytime**					
$PM_{2.5}$	$\mu g/m^3$	42.73±46.70	29.70 (19.58-47.16)	0.03	187.70
PM_{10}	$\mu g/m^3$	47.47±50.70	36.28 (21.53-51.74)	0.14	200.17
CO_2	PPM	850.65±374.67	843.47 (759.83-1058.23)	0.00	1386.94
HCHO	mg/m^3	0.03±0.02	0.02 (0.01-0.03)	0.01	0.08
TVOC	mg/m^3	0.33±0.16	0.33 (0.21-0.45)	0.11	0.64
Temperature	℃	23.92±4.39	24.38 (21.11-26.13)	16.87	33.60
Humidity	%	33.49±9.48	33.82 (25.43-39.44)	17.99	50.92
**Bedroom during weekdays daytime**					
$PM_{2.5}$	$\mu g/m^3$	43.01±55.21	26.71 (9.29-52.73)	0.00	404.96
PM_{10}	$\mu g/m^3$	47.63±61.04	29.58 (10.42-58.65)	0.02	437.10
CO_2	PPM	698.02±327.89	602.26 (495.99-795.90)	0.00	2007.90
HCHO	mg/m^3	0.02±0.05	0.01 (0.01-0.02)	0.00	0.43
TVOC	mg/m^3	0.33±0.15	0.32 (0.22-0.42)	0.10	0.81
Temperature	℃	25.59±3.35	25.31 (23.35-27.77)	13.63	33.50
Humidity	%	37.80±10.23	37.77 (30.56-44.89)	13.08	61.31
**Bedroom during weekdays nighttime**					
$PM_{2.5}$	$\mu g/m^3$	40.36±50.68	27.43 (10.00-50.96)	0.01	371.66
PM_{10}	$\mu g/m^3$	44.55±56.81	30.96 (11.28-55.94)	0.02	435.86
CO_2	PPM	1144.58±610.31	998.66 (709.95-1421.59)	0.00	3433.78
HCHO	mg/m^3	0.03±0.09	0.01 (0.01-0.03)	0.00	0.91
TVOC	mg/m^3	0.40±0.17	0.38 (0.27-0.50)	0.10	1.24
Temperature	℃	25.49±3.14	25.22 (23.29-27.58)	14.71	34.23
Humidity	%	41.38±9.83	40.58 (34.79-49.02)	15.6	63.85
**Bedroom during weekends daytime**					
$PM_{2.5}$	$\mu g/m^3$	46.03±67.99	23.63 (8.30-54.81)	0.42	570.32
PM_{10}	$\mu g/m^3$	51.15±75.78	25.51 (9.61-59.29)	0.55	621.29
CO_2	PPM	863.29±437.84	776.64 (553.79-1033.10)	0.00	2964.71
HCHO	mg/m^3	0.03±0.05	0.01 (0.01-0.03)	0.00	0.54
TVOC	mg/m^3	0.37±0.15	0.36 (0.26-0.46)	0.10	0.86
Temperature	℃	25.54±3.19	25.54 (23.45-27.17)	16.31	34.24
Humidity	%	38.60±10.16	37.35 (31.66-46.13)	19.09	64.01

**Bedroom during weekends nighttime**					
$PM_{2.5}$	μg/m^3	41.27±68.21	20.22 (9.02-50.55)	0.41	817.93
PM_{10}	μg/m^3	45.95±79.96	22.66 (10.04-53.65)	0.62	994.49
CO_2	PPM	1189.96±701.73	1000.23 (688.03-1544.20)	0.00	4481.49
HCHO	mg/m^3	0.03±0.08	0.01 (0.01-0.03)	0.00	0.93
TVOC	mg/m^3	0.43±0.18	0.42 (0.30-0.53)	0.10	1.00
Temperature	°C	25.36±3.14	25.38 (23.17-27.21)	15.86	33.63
Humidity	%	41.71±9.75	41.05 (35.04-48.37)	20.01	67.3

SD, standard deviation; Q_{25}, the 25th percentile; Q_{75}, the 75th percentile; Min, Minimum; Max, Maximum; $PM_{2.5}$, particulate matter with an aerodynamic diameter of ≤2.5 μm; PM_{10}, particulate matter with an aerodynamic diameter of ≤10 μm; CO_2, carbon dioxide; HCHO, formaldehyde; TVOC, total volatile organic compounds.

[a]For daytime, daily exposure was estimated by averaging concentrations from 8:00 to 17:00. For night, daily exposure was estimated by averaging concentrations from 17:00 to 8:00 the next morning. The final daily exposure was calculated by averaging the time-specific concentrations across the corresponding location–time combinations.

Table S4. Distributions of time-weighted overall exposure to seven indoor environmental factors among 219 children and adolescents in Beijing, by sex.

Exposure[a]	Units	Mean± SD	Median (Q_{25}-Q_{75})	Min	Max	Exceedance rate (%)[b]
**Boys**						
$PM_{2.5}$	μg/m^3	41.94±47.52	32.13 (14.04-52.19)	0.77	339.92	26.96
PM_{10}	μg/m^3	46.51±53.22	34.81 (16.01-57.48)	0.86	397.39	7.83
CO_2	PPM	1012.10±454.74	928.89 (699.47-1269.59)	0.00	2723.69	41.74
HCHO	mg/m^3	0.04±0.10	0.02 (0.01-0.03)	0.00	1.09	5.22
TVOC	mg/m^3	0.38±0.13	0.36 (0.29-0.45)	0.12	0.76	7.83
Temperature	℃	25.39±3.17	25.24 (22.99-27.23)	14.99	33.99	31.30
Humidity	%	39.75±9.22	39.39 (32.82-47.80)	18.63	57.59	33.04
**Girls**						
$PM_{2.5}$	μg/m^3	39.86±49.93	28.67 (10.67-51.26)	0.59	383.21	27.88
PM_{10}	μg/m^3	44.32±57.50	32.41 (11.85-55.99)	0.79	453.3	5.77
CO_2	PPM	1070.76±528.40	1029.58 (733.28-1335.55)	0	3025.33	53.85
HCHO	mg/m^3	0.03±0.03	0.02 (0.01-0.03)	0.01	0.15	7.69
TVOC	mg/m^3	0.40±0.14	0.40 (0.29-0.49)	0.12	0.77	9.62
Temperature	℃	25.29±3.26	24.95 (23.26-27.97)	17.67	32.94	39.42
Humidity	%	39.98±8.68	39.40 (33.90-46.65)	21.02	56.57	35.58

SD, standard deviation; Q_{25}, the 25th percentile; Q_{75}, the 75th percentile; Min, Minimum; Max, Maximum; $PM_{2.5}$, particulate matter with an aerodynamic diameter of ≤2.5 μm; PM_{10}, particulate matter with an aerodynamic diameter of ≤10 μm; CO_2, carbon dioxide; HCHO, formaldehyde; TVOC, total volatile organic compounds.

[a]Indoor exposure was calculated as a time-weighted average of classroom and bedroom concentrations. For weekdays, daily exposure was estimated by averaging classroom concentrations from 08:00 to 17:00 and bedroom concentrations from 17:00 to 8:00 the next morning. The final combined exposure was obtained by averaging the daily values across the entire week.

[b]The exceedance rate for each subgroup was calculated as the proportion of samples within that subgroup that exceeded the recommended limits defined in the Indoor Air Quality Standard (GB/T 18883–2022) of China.

Table S5. Distributions of time-weighted overall exposure to seven indoor environmental factors among 219 children and adolescents in Beijing, by grade.

Exposure[a]	Units	Mean± SD	Median (Q_{25}-Q_{75})	Min	Max	Exceedance rate (%)[b]
**Grades 1-4**						
$PM_{2.5}$	μg/m³	37.02±28.44	34.92 (12.85-52.99)	0.59	137.66	29.66
PM_{10}	μg/m³	40.73±31.01	38.77 (14.77-58.98)	0.79	149.79	4.14
CO_2	PPM	970.35±456.72	899.36 (619.13-1218.70)	0.00	2740.45	40.69
HCHO	mg/m³	0.03±0.02	0.02 (0.01-0.03)	0.00	0.15	4.14
TVOC	mg/m³	0.39±0.13	0.37 (0.29-0.46)	0.12	0.77	7.59
Temperature	℃	25.69±2.77	25.42 (23.79-27.22)	18.01	33.99	29.66
Humidity	%	42.07±8.76	42.49 (36.75-49.23)	18.63	56.72	29.66
**Grades 7**						
$PM_{2.5}$	μg/m³	48.65±73.22	26.14 (12.92-45.93)	1.65	383.21	22.97
PM_{10}	μg/m³	54.76±84.04	28.55 (15.03-50.45)	1.83	453.3	12.16
CO_2	PPM	1176.34±528.67	1141.07 (861.86-1453.04)	0.00	3025.33	60.81
HCHO	mg/m³	0.05±0.13	0.01 (0.01-0.04)	0.01	0.72	10.81
TVOC	mg/m³	0.39±0.15	0.38 (0.30-0.48)	0.12	1.09	10.81
Temperature	℃	24.66±3.86	23.98 (21.99-28.22)	14.99	32.14	45.95
Humidity	%	35.54±7.68	34.81 (30.22-39.83)	21.02	57.59	43.24

SD, standard deviation; Q_{25}, the 25th percentile; Q_{75}, the 75th percentile; Min, Minimum; Max, Maximum; $PM_{2.5}$, particulate matter with an aerodynamic diameter of ≤2.5 μm; PM_{10}, particulate matter with an aerodynamic diameter of ≤10 μm; CO_2, carbon dioxide; HCHO, formaldehyde; TVOC, total volatile organic compounds.

[a]Indoor exposure was calculated as a time-weighted average of classroom and bedroom concentrations. For weekdays, daily exposure was estimated by averaging classroom concentrations from 08:00 to 17:00 and bedroom concentrations from 17:00 to 8:00 the next morning. The final combined exposure was obtained by averaging the daily values across the entire week.

[b]The exceedance rate for each subgroup was calculated as the proportion of samples within that subgroup that exceeded the recommended limits defined in the Indoor Air Quality Standard (GB/T 18883–2022) of China.

Table S6. Distributions of time-weighted overall exposure to seven indoor environmental factors among 219 children and adolescents in Beijing, by maternal education.

Exposure[a]	Units	Mean± SD	Median (Q_{25}-Q_{75})	Min	Max	Exceedance rate (%)[b]
**Higher education**						
$PM_{2.5}$	μg/m^3	31.36±39.43	22.32 (10.72-40.12)	0.59	339.92	16.49
PM_{10}	μg/m^3	34.95±45.21	22.02 (12.69-43.56)	0.79	397.39	3.09
CO_2	PPM	1013.57±449.73	954.48 (704.89-1243.91)	0.00	2549.06	45.36
HCHO	mg/m^3	0.03±0.03	0.02 (0.01-0.03)	0.00	0.27	4.12
TVOC	mg/m^3	0.39±0.14	0.37 (0.31-0.47)	0.12	0.77	8.00
Temperature	℃	24.94±3.01	24.51 (22.96-26.87)	14.99	33.99	31.96
Humidity	%	38.82±8.69	38.37 (32.08-45.65)	18.63	56.47	36.08
**Lower education**						
$PM_{2.5}$	μg/m^3	52.93±60.96	40.61 (15.25-63.45)	1.65	383.21	37.33
PM_{10}	μg/m^3	58.55±68.97	44.64 (16.37-70.27)	1.83	453.3	13.33
CO_2	PPM	1087.93±555.57	1053.79 (694.55-1384.71)	0.00	3025.33	53.33
HCHO	mg/m^3	0.04±0.13	0.02 (0.01-0.04)	0.01	0.72	9.33
TVOC	mg/m^3	0.40±0.13	0.40 (0.28-0.45)	0.16	1.09	9.28
Temperature	℃	25.53±3.70	25.40 (22.75-28.42)	17.67	32.94	41.33
Humidity	%	39.57±9.07	39.39 (32.48-46.95)	22.86	57.59	34.67

SD, standard deviation; Q_{25}, the 25th percentile; Q_{75}, the 75th percentile; Min, Minimum; Max, Maximum; $PM_{2.5}$, particulate matter with an aerodynamic diameter of ≤2.5 μm; PM_{10}, particulate matter with an aerodynamic diameter of ≤10 μm; CO_2, carbon dioxide; HCHO, formaldehyde; TVOC, total volatile organic compounds.

[a]Indoor exposure was calculated as a time-weighted average of classroom and bedroom concentrations. For weekdays, daily exposure was estimated by averaging classroom concentrations from 08:00 to 17:00 and bedroom concentrations from 17:00 to 8:00 the next morning. The final combined exposure was obtained by averaging the daily values across the entire week. Cool season was defined as November to March, while warm season was defined as April to October.

[b]The exceedance rate for each subgroup was calculated as the proportion of samples within that subgroup that exceeded the recommended limits defined in the Indoor Air Quality Standard (GB/T 18883–2022) of China.

Table S7. Distributions of time-weighted overall exposure to seven indoor environmental factors among 219 children and adolescents in Beijing, by decoration.

Exposure[a]	Units	Mean± SD	Median (Q_{25}-Q_{75})	Min	Max	Exceedance rate (%)[b]
**Yes**						
$PM_{2.5}$	μg/m^3	41.46±44.79	30.27 (14.77-54.82)	1.09	339.92	27.45
PM_{10}	μg/m^3	46.29±51.47	32.96 (15.96-61.73)	1.48	397.39	6.86
CO_2	PPM	1137.02±555.38	1018.13 (824.83-1320.63)	0.00	3025.33	50.98
HCHO	mg/m^3	0.03±0.02	0.02 (0.01-0.03)	0.01	0.27	8.00
TVOC	mg/m^3	0.38±0.13	0.38 (0.28-0.44)	0.12	0.68	9.26
Temperature	℃	24.58±3.01	24.40 (22.52-26.60)	14.99	32.94	26.67
Humidity	%	39.37±9.05	39.02 (31.09-46.94)	22.86	57.59	29.33
**No**						
$PM_{2.5}$	μg/m^3	40.51±52.20	28.46 (11.79-48.82)	0.59	383.21	26.85
PM_{10}	μg/m^3	44.90±59.04	32.22 (14.05-52.86)	0.79	453.3	6.48
CO_2	PPM	993.85±459.96	940.08 (614.81-1302.56)	0.00	2190.28	43.52
HCHO	mg/m^3	0.03±0.03	0.02 (0.01-0.03)	0.00	0.15	4.62
TVOC	mg/m^3	0.40±0.14	0.36 (0.29-0.47)	0.13	0.77	8.82
Temperature	℃	25.73±3.27	25.48 (23.42-27.99)	17.67	33.99	39.39
Humidity	%	40.01±9.02	39.57 (34.02-47.73)	18.63	56.47	37.88

SD, standard deviation; Q_{25}, the 25th percentile; Q_{75}, the 75th percentile; Min, Minimum; Max, Maximum; $PM_{2.5}$, particulate matter with an aerodynamic diameter of ≤2.5 μm; PM_{10}, particulate matter with an aerodynamic diameter of ≤10 μm; CO_2, carbon dioxide; HCHO, formaldehyde; TVOC, total volatile organic compounds.

[a]Indoor exposure was calculated as a time-weighted average of classroom and bedroom concentrations. For weekdays, daily exposure was estimated by averaging classroom concentrations from 08:00 to 17:00 and bedroom concentrations from 17:00 to 8:00 the next morning. The final combined exposure was obtained by averaging the daily values across the entire week. Cool season was defined as November to March, while warm season was defined as April to October.

[b]The exceedance rate for each subgroup was calculated as the proportion of samples within that subgroup that exceeded the recommended limits defined in the Indoor Air Quality Standard (GB/T 18883–2022) of China.

Table S8. Distributions of time-weighted overall exposure to seven indoor environmental factors among 219 children and adolescents in Beijing, by season.

Exposure[a]	Units	Mean± SD	Median (Q_{25}-Q_{75})	Min	Max	Exceedance rate (%)[b]
**Warm**						
$PM_{2.5}$	μg/m^3	44.55±50.65	27.80 (11.93-61.78)	3.82	310.17	33.8
PM_{10}	μg/m^3	48.52±55.55	29.16 (14.22-65.46)	4.42	334.26	8.45
CO_2	PPM	705.62±385.27	552.95 (483.05-921.25)	0.00	1986.85	15.49
HCHO	mg/m^3	0.05±0.13	0.02 (0.01-0.03)	0.01	0.68	7.04
TVOC	mg/m^3	0.36±0.13	0.35 (0.27-0.42)	0.16	1.09	8.26
Temperature	℃	28.71±1.83	28.33 (27.13-30.14)	26.38	33.99	57.75
Humidity	%	42.21±6.63	40.36 (36.84-48.30)	32.38	56.72	49.30
**Cool**						
$PM_{2.5}$	μg/m^3	41.95±56.54	29.14 (12.14-46.20)	0.59	383.21	22.45
PM_{10}	μg/m^3	47.44±65.63	32.86 (15.03-49.64)	0.79	453.3	9.18
CO_2	PPM	1281.22±487.12	1169.14 (964.53-1481.12)	523.2	3025.33	72.45
HCHO	mg/m^3	0.02±0.02	0.01 (0.01-0.03)	0.00	0.14	6.12
TVOC	mg/m^3	0.41±0.15	0.41 (0.31-0.48)	0.12	0.77	9.18
Temperature	℃	22.99±2.42	22.93 (21.75-24.22)	14.99	30.76	33.67
Humidity	%	35.86±9.28	34.71 (28.11-41.93)	18.63	57.59	28.57

SD, standard deviation; Q_{25}, the 25th percentile; Q_{75}, the 75th percentile; Min, Minimum; Max, Maximum; $PM_{2.5}$, particulate matter with an aerodynamic diameter of ≤2.5 μm; PM_{10}, particulate matter with an aerodynamic diameter of ≤10 μm; CO_2, carbon dioxide; HCHO, formaldehyde; TVOC, total volatile organic compounds.

[a]Indoor exposure was calculated as a time-weighted average of classroom and bedroom concentrations. For weekdays, daily exposure was estimated by averaging classroom concentrations from 08:00 to 17:00 and bedroom concentrations from 17:00 to 8:00 the next morning. The final combined exposure was obtained by averaging the daily values across the entire week. Cool season was defined as November to March, while warm season was defined as April to October.

[b]The exceedance rate for each subgroup was calculated as the proportion of samples within that subgroup that exceeded the recommended limits defined in the Indoor Air Quality Standard (GB/T 18883–2022) of China.

Table S9. Characteristics of 130 study participants with and without depressive symptoms.

Characteristic	Overall (N =130)	With depressive symptoms (N =34)	Without depressive symptoms (N =96)	P value
**Demographical factors**				
Age (years), mean ± SD	11.6 ± 1.9	12.4 ± 1.7	11.2 ± 1.9	0.003
Grade, n (%)				
Grades 3-4	68 (52.3)	12 (35.3)	56 (58.3)	0.04
Grade 7	62 (47.7)	22 (64.7)	40 (41.7)	
Sex, n (%)				
Boys	62 (47.7)	14 (41.2)	48 (50.0)	0.50
Girls	68 (52.3)	20 (58.8)	48 (50.0)	
Maternal Education, n (%)				
Bachelor's degree or above	74 (56.9)	14 (41.2)	60 (62.5)	0.05
High school or below	56 (43.1)	20 (58.8)	36 (37.5)	
Only child status, n (%)				
Yes	93 (71.5)	20 (58.8)	73 (76.0)	0.09
No	37 (28.5)	14 (41.2)	23 (24.0)	
**Having an unfavorable lifestyle, n (%)**				
Insufficient sleep[a]	10 (7.7)	2 (5.9)	8 (8.3)	0.931
Failing to meet fruit/vegetable and protein intake (within past 7 days)[b]	92 (70.8)	29 (85.3)	63 (65.6)	0.05
Consuming SSBs (within past 7 days)	34 (26.2)	8 (23.5)	26 (27.1)	0.86
Low physical activity[c]	60 (46.2)	14 (41.2)	46 (47.9)	0.63
Recreational screen time≥2 h per day	130 (100.0)	34 (100.0)	96 (100.0)	/
**Number of unfavorable lifestyle factors, n (%)**				0.92
0–2 (favourable lifestyle)	14 (10.8)	3 (8.8)	11 (11.5)	
3–5 (favourable lifestyle)	116 (89.2)	31 (91.2)	85 (88.5)	
**Experiencing peer bullying, n (%)**				
Yes	36 (27.7)	15 (44.1)	21 (21.9)	0.02
No	94 (72.3)	19 (55.9)	75 (78.1)	
**Season at the time of indoor monitoring, n (%)**				

Warm	49 (37.7)	13 (38.2)	36 (37.5)	0.93
Cold	81 (62.3)	21 (61.8)	60 (62.5)	
**Primary ventilation method, n (%)**				
Window	120 (92.3)	33 (97.1)	87 (90.6)	0.40
Machine	10 (7.7)	1 (2.9)	9 (9.4)	
**Recent home renovation, n (%)**				
Yes	49 (37.7)	20 (58.8)	29 (30.2)	
No	74 (56.9)	13 (38.2)	61 (63.5)	0.01
Unknown	7 (5.4)	1 (2.9)	6 (6.2)	
**Regular use of air purifiers, n (%)**				
Yes	59 (45.4)	19 (55.9)	40 (41.7)	0.22
No	71 (54.6)	15 (44.1)	56 (58.3)	

SSB, Sugar-sweetened beverage; SD, standard deviation; BMI, body mass index; WHO, World Health Organization; WC, Waist circumference; FBG, fasting glucose; TG, triglycerides; SBP, systolic blood pressure; HDL-C, high density lipoprotein cholesterol; SSB, Sugar-sweetened beverages.

[a]Insufficient sleep was categorized as <10 h per day for primary school students, <9 h per day for junior high school students, according to the Healthy China Action Plan.

[b]Days consumed 5 servings of fruits/vegetables and 1 serving of high-quality protein (past 7 days).

[c]Total physical activity levels (metabolic equivalent task-hours per day, MET-h/d) were calculated by multiplying hours spent on each activity per day by corresponding physical intensity and then summing up the MET-h/d for all activities. Participants were classified into low and high physical activity levels based on sex- and age-specific median cutoffs.

Table S10. Associations between exposure to indoor air pollutants and CES-D scores among children and adolescents.

Exposure[a]	Increment (IQR)	*β* (95% CIs)[b]	*P* values
$PM_{2.5}$	39.44 μg/m^3	-0.32 (-1.68, 1.03)	0.64
PM_{10}	43.83 μg/m^3	-0.30 (-1.59, 0.99)	0.65
CO_2	552.38 PPM	5.17 (2.35, 8.00)	<0.001*
HCHO	0.02 mg/m^3	0.59 (-0.73, 1.92)	0.46
TVOC	0.21 mg/m^3	2.91 (0.23, 5.59)	0.03*

$PM_{2.5}$, particulate matter with an aerodynamic diameter of ≤2.5 μm; PM_{10}, particulate matter with an aerodynamic diameter of ≤10 μm; CO_2, carbon dioxide; HCHO, formaldehyde; TVOC, total volatile organic compounds. CES-D, Center for Epidemiologic Studies Depression Scale; CI, confidence interval.

[a]Indoor exposure was calculated as a time-weighted average of classroom and bedroom concentrations. For weekdays, daily exposure was estimated by averaging classroom concentrations from 08:00 to 17:00 and bedroom concentrations from 17:00 to 8:00 the next morning. For weekends, daily exposure was estimated using 24-hour bedroom concentrations. The final combined exposure was obtained by averaging the daily values across the entire week.

[b]Effect estimates were coefficients of CES-D scores associated with an interquartile increase in indoor environmental exposure. Associations were adjusted for a common set of confounders: age, sex, maternal education, number of unfavorable lifestyle factors, peer bullying, indoor temperature and relative humidity, recent home renovation, primary ventilation method, use of air purifiers and season at the time of indoor monitoring. Factors listed in the confounder set were adjusted for each other.

*Two-tail significance level was set at $p < 0.05$.

Table S11. Associations between exposure to indoor air pollutants and depressive symptoms (using CES-D scores ≥16) among children and adolescents.

Exposure[a]	Increment (IQR)	ORs (95%CIs)[b]	*P* values
$PM_{2.5}$	39.44 μg/m^3	0.97 (0.69, 1.38)	0.87
PM_{10}	43.83 μg/m^3	0.98 (0.70, 1.37)	0.92
CO_2	552.38 PPM	2.67 (1.41, 5.06)	0.002*
HCHO	0.02 mg/m^3	1.16 (0.84, 1.62)	0.36
TVOC	0.21 mg/m^3	1.73 (1.02, 2.91)	0.03*

$PM_{2.5}$, particulate matter with an aerodynamic diameter of ≤2.5 μm; PM_{10}, particulate matter with an aerodynamic diameter of ≤10 μm; CO_2, carbon dioxide; HCHO, formaldehyde; TVOC, total volatile organic compounds. OR, odds ratio; CI, confidence interval.

[a]Indoor exposure was calculated as a time-weighted average of classroom and bedroom concentrations. For weekdays, daily exposure was estimated by averaging classroom concentrations from 08:00 to 17:00 and bedroom concentrations from 17:00 to 8:00 the next morning. For weekends, daily exposure was estimated using 24-hour bedroom concentrations. The final combined exposure was obtained by averaging the daily values across the entire week.

[b]Effect estimates were odds ratios of depressive symptoms associated with an interquartile increase inindoor environmental exposure. Associations were adjusted for a common set of confounders: age, sex, maternal education, number of unfavorable lifestyle factors, peer bullying, indoor temperature and relative humidity, recent home renovation, primary ventilation method, use of air purifiers and season at the time of indoor monitoring. Factors listed in the confounder set were adjusted for each other.

*Two-tail significance level was set at $p < 0.05$.

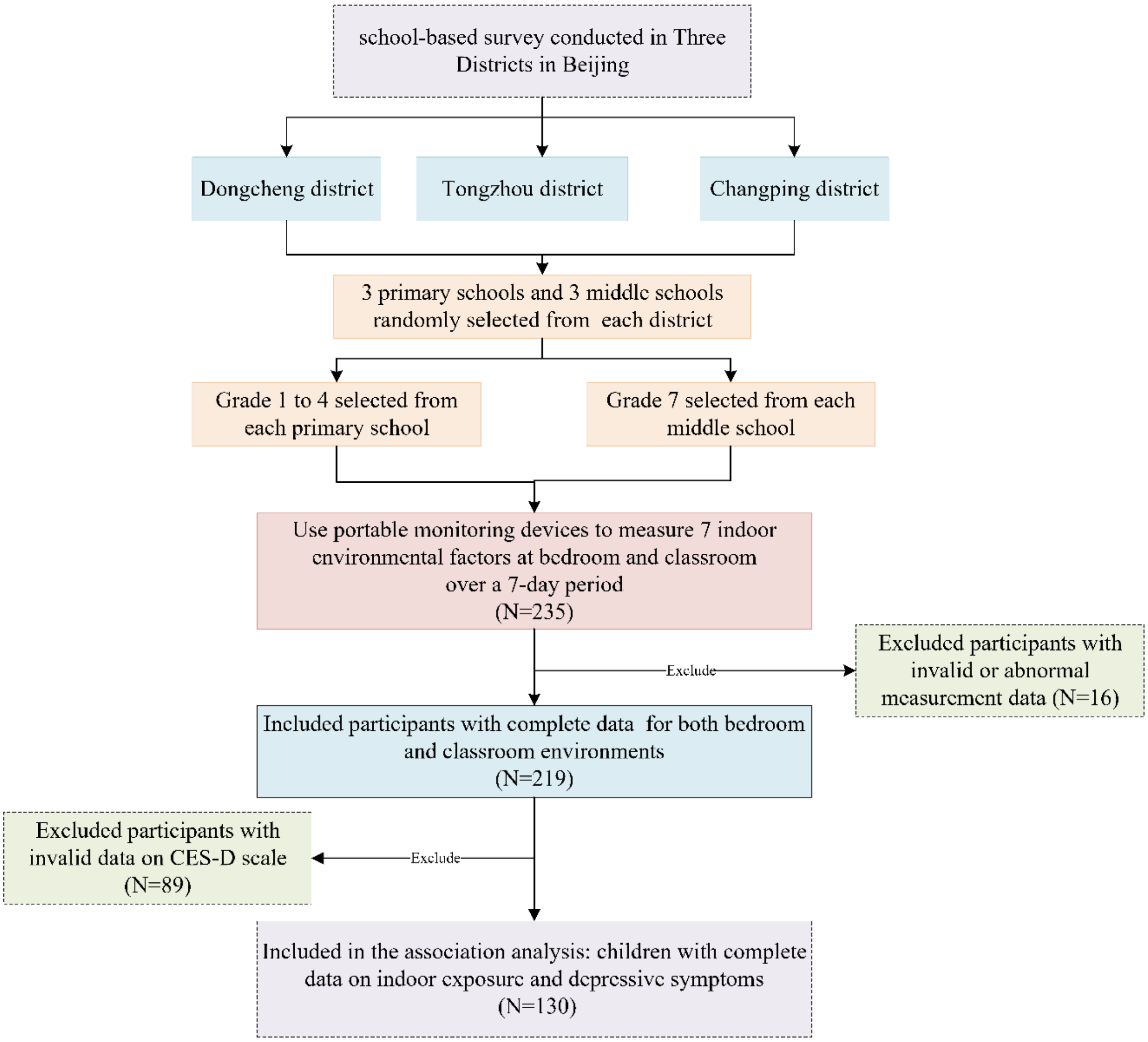

Figure S1. Flowchart of participants' inclusion.

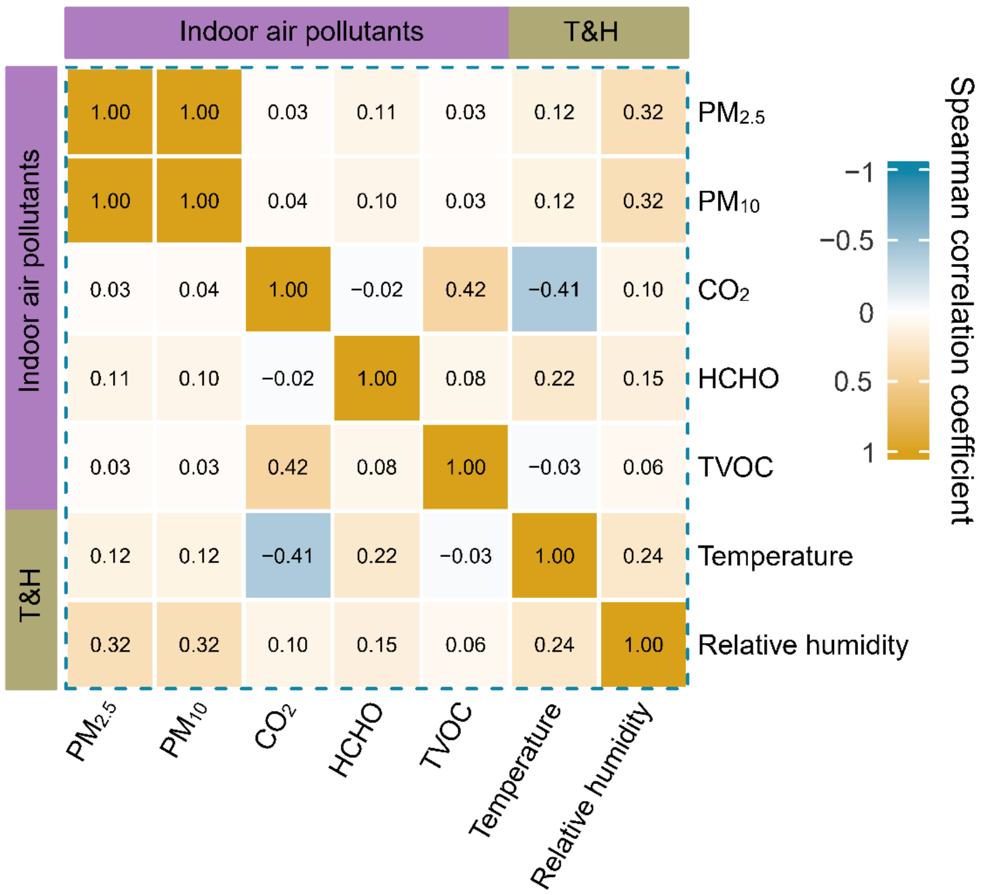

Figure S2. Spearman correlation coefficients between indoor environmental exposures based on weighted average concentrations. $PM_{2.5}$, particulate matter with an aerodynamic diameter of ≤2.5 μm; PM_{10}, particulate matter with an aerodynamic diameter of ≤10 μm; CO_2, carbon dioxide; HCHO, formaldehyde; TVOC, total volatile organic compounds.

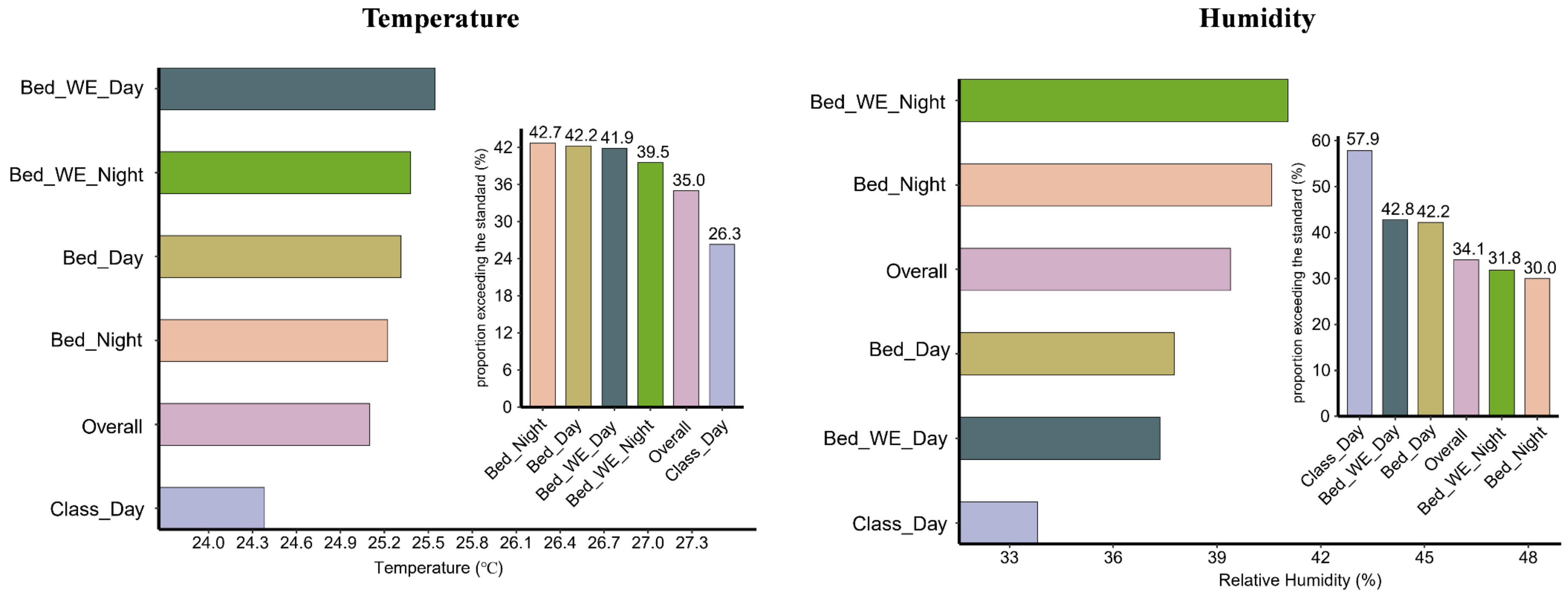

Figure S3. Median levels and exceedance rates of two indoor environmental parameters across different exposure settings and time periods. Horizontal bar charts represent the median values of two indoor environmental parameters (temperature and relative humidity) across various indoor exposure settings and time periods, including weekday/weekend and classroom/bedroom contexts. Vertical bar charts show the corresponding proportions of samples exceeding the recommended limits defined in the Indoor Air Quality Standard (GB/T 18883–2022) of China. Class_Day, classroom during weekday daytime, Bed_Day, bedroom during weekday daytime, Bed_Night, bedroom during weekday nighttime, Bed_WE_Day, bedroom during weekend day-time, Bed_WE_Night, bedroom during weekend nighttime. “Overall” represents the average across all time periods.

Supplemental Questionnaire

1. Does the child's fother currently smoke?

[1] Yes, smokes every day (Answer question 1.1)

[2] Yes, but not every day (Answer question 1.2)

[3] Does not smoke (Skip to question 2)

1.1. On average, how many cigarettes does he smoke per day?

Answer: ___ sticks/day

1.2. On average, how many cigarettes does he smoke per week?

Answer: ___ sticks/week

2. Has the child's father used e-cigarettes in the past six months?

[1] Yes

[2] No (Skip to question 7)

2.1. On average, how many days per month does the child's father come into contact with e-cigarettes?

[1] Less than once

[2] 1–2 days

[3] 3–5 days

[4] 6–9 days

[5] 10–19 days

[6] 20–29 days

[7] Almost every day

2.2. On a typical day when he uses e-cigarettes, how many times does he use them?

Answer: ___ times/day

Example: If he uses e-cigarettes on two days per week, please calculate the average number of uses per day on those days.

3. Does the child's mother currently smoke?

[1] Yes, smokes every day (Answer question 3.1)

[2] Yes, but not every day (Answer question 3.2)

[3] Does not smoke (Skip to question 4)

3.1. On average, how many cigarettes does she smoke per day?

Answer: ___ sticks/day

3.2. On average, how many cigarettes does she smoke per week?

Answer: ___ sticks/week

4. Has the child's mother used e-cigarettes in the past six months?

[1] Yes

[2] No (Skip to question 5)

4.1. On average, how many days per month does the child's mother use e-cigarettes?
[1] Less than once
[2] 1–2 days
[3] 3–5 days
[4] 6–9 days
[5] 10–19 days
[6] 20–29 days
[7] Almost every day

4.2. On a typical day when he uses e-cigarettes, how many times does she use them?
Answer: ___ times/day
Example: If he uses e-cigarettes on two days per week, please calculate the average number of uses per day on those days.
